# Prolonged Transcriptional Consequences in Survivors of Sepsis

**DOI:** 10.3390/ijms22115422

**Published:** 2021-05-21

**Authors:** Krzysztof Laudanski, James Soh, Matthew DiMeglio, Kathleen E. Sullivan

**Affiliations:** 1 Department of Anesthesiology and Critical Care, The University of Pennsylvania, Philadelphia, PA 19104, USA; 2 Department of Neurology, The University of Pennsylvania, Philadelphia, PA 19104, USA; sohja@pennmedicine.upenn.edu; 3 Leonard Davis Institute of Healthcare Economics, The University of Pennsylvania, Philadelphia, PA 19104, USA; 4 School of Medicine, Jefferson University, Philadelphia, PA 19104, USA; Matthew.DiMeglio@jefferson.edu; 5 Division of Allergy and Immunology, Children Hospital Philadelphia, Philadelphia, PA 19104, USA; sullivank@email.chop.edu

**Keywords:** sepsis, post-septic syndrome, bacteremia, gram-negative, survival, long-term, genomic profiling, RNA-seq, miRNA, metabolomic, glutathione pathway, *Drosophila melanogaster*

## Abstract

Survivors of sepsis often suffer from prolonged post-critical illness syndrome secondary to the immune system’s reprogramming. It is unclear if this process is static and pervasive due to methodological difficulties studying long-term outcomes of sepsis. The purpose of this study is to evaluate transcriptional profiles longitudinally in *Drosophila melanogaster* in the aftermath of sepsis to provide preliminary data for targets playing a role in post-sepsis immunostasis. Adult *Drosophila melanogaster* were infected with *E. coli*, and survivors were euthanized at 7, 14, and 21 days. Control flies were subjected to sham stress. Gene profiling was done with RNA-seq, and potential miRNA factors were computed. Profiling identified 55 unique genes at seven days, 61 unique genes at 14 days, and 78 genes at 21 days in sepsis survivors vs. sham control. Each post-sepsis timepoint had a distinctive transcriptional pattern with a signature related to oxidative stress at seven days, neuronal signal transduction at 14 days, and metabolism at 21 days. Several potential miRNA patterns were computed as potentially affecting several of the genes expressed in sepsis survivors. Our study demonstrated that post-sepsis changes in the transcriptome profile are dynamic and extend well into the *Drosophila melanogaster* natural life span.

## 1. Introduction

Under ideal conditions, sepsis eliminates the offending pathogen without significant collateral damage [1,2]. The immune system should refocus on repair and regeneration and eventually de-activate. This intricate process can be derailed, resulting in the host’s demise secondary to excessive, abnormal, persistent, or ineffective immune system activation [3,4,5,6]. 

The completeness of the resolution of sepsis and recovery of health has been questioned [6,7]. Experimental studies suggest a metabolome and immune system being affected in the long term [8,9,10]. Epidemiological studies demonstrate progressive organ damage and altered metabolism in sepsis survivors [3,11,12]. Pervasive increase in mortality is likely secondary to the emergence of alternative states of immunostasis that are distinctive from pre-insult health in victims of sepsis [4,5]. Mechanisms affecting access to DNA information (epigenetic, non-coding RNA, miRNA) were suggested to maintain the post-sepsis reprogramming [1,2,8,9,10,13,14]. 

There is a gap in knowledge of how gene reprogramming evolves in sepsis survivors. This is both an essential basic science and clinical management question [6,7]. Recovery from sepsis is likely to be as heterogenous as sepsis itself, particularly when temporal and longitudinal changes are considered. The considerable prevalence of sepsis, COVID-19 pandemic, and increasing survivorship render studies investigating sepsis’s long-term consequence of paramount clinical and societal importance [4,6,7,11]. Unfortunately, longitudinal studies are inherently difficult [6,7]. Long-term enrollment of human subjects is complicated by the unknown pre-sepsis immunological and transcriptional makeup, high inter-individual heterogeneity, and diverse clinical presentation of the disease [6]. Furthermore, studying the effect of sepsis or progression of the natural process of aging is inherently difficult as both processes are interconnected and affected by several variables [15,16]. Prior research demonstrated the emergence and, at minimum short-term persistence of several metabolomic and immunological abnormalities in the peri-septic period.

In order to overcome some of the methodological difficulties, several alternative models of sepsis have been proposed [6,17]. Non-human models were utilized to demonstrate several fundamental processes underpinning sepsis emergence and recovery [18]. *Drosophila* species have been a pivotal model for several essential research findings, including the discovery of toll-like receptors [19,20]. Sepsis can be induced in *Drosophila* using lipopolysaccharide exposure or bacterial inoculation, providing critical information about the pathogen interacting with the host [21,22,23,24,25]. Therefore, sepsis in fruit flies is defined by most researchers as the exposure itself to a sepsis trigger, not by physiological parameters as seen in clinical settings [6]. However, the effect of sepsis and bacteremia seems to be similar in patients, suggesting that interaction with a pathogen is critical for host performance, not downstream effects of sepsis [26,27,28]. Several highly conservative pathways (p38, NFκB, Rel, ubiquitins) were of particular interest in *Drosophila* sepsis [21,29,30]. Monocytes, a critical component of sepsis response, showed typical sepsis polarization and cytokine activation in *Drosophila* [31]. In particular, the downstream activation of the interleukin-1 family closely resembles events seen in clinical settings [2,17,21]. Finally, metabolomic responses were studied, demonstrating similar abnormalities as those seen in clinical sepsis [32]. Therefore, it is not a surprise that several Drosophila-based studies translated into successful studies in more clinically oriented settings, replicated studies from animal models of sepsis or observation from humans [22,24,25]. There is increased appreciation for *Drosophila* as a sepsis model, coupled with its potential to address several inherently difficult questions in sepsis responses. A shorter life span of the fly provides a convenient insight into the sepsis effects on aging. Experiments may be scaled easily and controlled for several variables, a frequent suggestion in sepsis research [1,6,7]. Finally, the effect of sepsis on embryonal life can be studied [33]. Nevertheless, the *Drosophila* model of sepsis seems to be underutilized given its potential.

Our study sought to investigate the transcriptome in survivors of sepsis using *Drosophila melanogaster* inoculated with *Escherichia coli.* We hypothesized that post-sepsis, the reprogramming measured as transcriptional activity would reflect important pathways critical for prolonged alteration of immune homeostasis after sepsis.

## 2. Results

### 2.1. Survivorship and Morphological Changes in Survivors of the Sepsis

We inoculated 60 flies with *Escherichia coli* (C_0) and observed the survivorship compared to sham-operated insects. We noticed significant attrition in survivors, particularly at 14 and 21 days as compared to sham surgery group. Additionally, visual inspection of flies showed much smaller insects (data not provided; personal observation) among survivors.

### 2.2. RNA Profile in the Sepsis Survivors at 7, 14, and 21 Days

**Figure 1:**
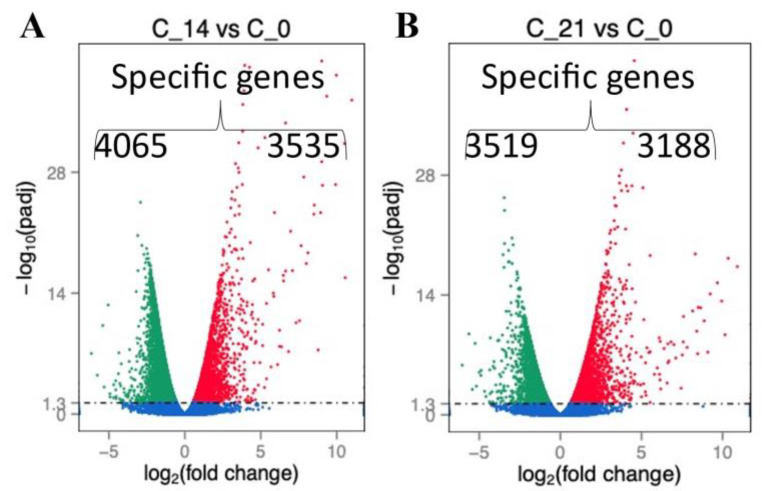
Distribution and number of genes that were differentially expressed at 14 (C_14) (**A**) and 21 (C_21) (**B**) days as compared to animals without surgery (C_0).

**Figure 2:**
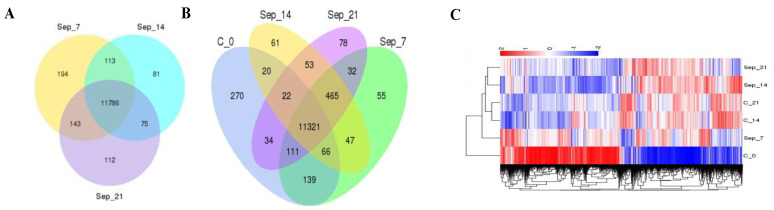
Sham surgery resulted in changes in gene expression over time (**A**), which was to a certain degree observed in sepsis survivors (**B**). Distinctive gene expression was seen when comparing flies surviving 21 days after sepsis compared to one and two week survivors or control animals. Unique gene patterns at 7, 14, and 21 among sepsis survivors can be seen (**C**).

We analyzed gene expression over time after the sham procedure at 14 and 21 days. We found several genes differentially expressed at both time points (Figure 1A,B and Figure 2A). Consequently, we age-matched flies post-sepsis and post-sham to account for the post-sepsis time effect. The transcriptional profiling indicated that flies at 14 days and 21 days post-sepsis had significant changes to their transcriptomes compared to the sham-treated flies (Figure 2B,C). While the flies are closer to controls at 21 days than at 14 days, there are still highly significant differences in gene expression (Figure 2C). We identified 77 unique genes for early post-sepsis recovery (d7), 61 unique genes for middle recovery (d14), and 78 genes specific for late recovery (d21) (Figure 2B). 

**Figure 3:**
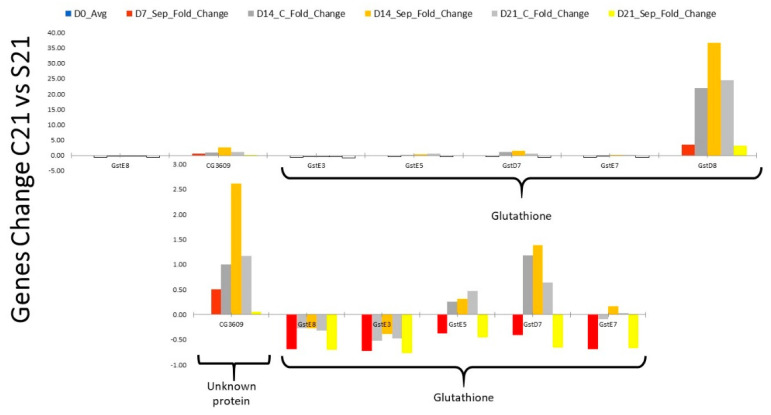
Abnormal expression of glutathione gene among Drosophila surviving sepsis is the dominant trait seen in our study.

Analysis of the most dominant genes revealed that specific pathways were affected during different recovery times from sepsis. 14 days after surgery, the predominant genes affected were related to the neurotransmitter-related genes (Supplementary Figure S1). At 21 days, most of the genes were related to metabolomic and glutathione pathways, in particular (Supplementary Figure S2; Figure 3). 

**Figure 4:**
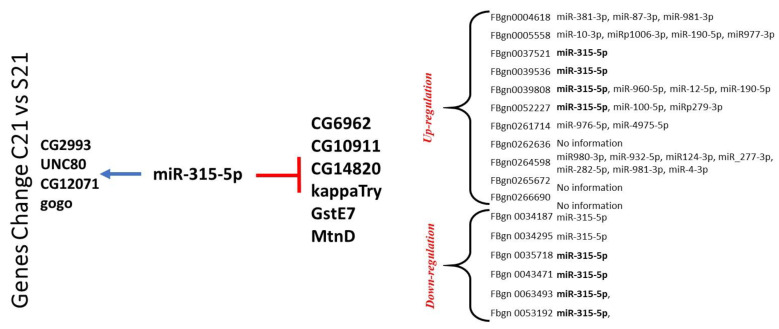
All of the genes analyzed had potential targeting sites by different microRNAs. The frequency of miR-315-5p in the gene list was highest and, notably, evolutionarily conserved among multiple Drosophila species unlike any other microRNAs studied. The involvement in miR-315-5p suggests several potential targets.

In addition to analyzing potential pathways and biological processes, we also examined the 3′ untranslated regions of all of the mRNAs (or genes) for the gene with altered expression at 14 and 21 days after sepsis. Five of the upregulated genes at 21 days showed potential targeting by miR-315-5p in an evolutionarily conserved manner [34]. In the group of downregulated genes, most did not reveal any evolutionarily conserved targeting by microRNAs in the 3′ untranslated region over multiple Drosophila variants. In the few that did, one gene, MtnD or metallothionein D, has a conserved binding spot for miR-315-5p; 6 other genes demonstrated a potential binding site for miR-315-5p but did not exhibit conservation over multiple Drosophila variants (Figure 4).

## 3. Discussion

Our results demonstrate that recovery from sepsis in *Drosophila melanogaster* is associated with specific transcriptional profiles that are time-dependent and distinct. This was suggested in prior work but not demonstrated [10]. Subsequent studies of RNA-seq expression showed several common findings [24]. As evidenced by the sheer number of genes studied, the effect of sepsis in *Drosophila* may have played an independent role in the decreased number of genes noted in later time points. Our report provides several potential suggestions to further investigate the mechanism-related to long-term abnormalities seen in sepsis survivors. 

The metabolic signature at 21 days was related to the glutathione pathway, a critical pathway for detoxification, free radical prevention, sepsis, and aging processes [35,36]. The glutathione pathway is essential for the elimination of pathogens while protecting the host from collateral damage [36,37]. GstD8 is also involved in the activation of p38 and JNK kinases, essential elements of immune response [38,39]. This interaction could be the leading pathway to neurotoxicity and shortened survival in a free radicals-rich environment, as seen during and post-sepsis [37,40,41]. Furthermore, impairment of glutathione pathways leads to increased susceptibility to infection and is a hallmark of abnormal resolution of inflammation [5,6,21,28,41,42]. Several of these pathways are activated at the beginning of the infection or clean injury [24]. Persistence of these pathways after resolution of the bacterial inoculation suggests metabolomic reprogramming [6,17,24]. Activation of the ubiquitination pathway affects the survival of Drosophila and may be responsible for cachexia and increased mortality in our study [21,36,40]. One of the future directions of research is to establish activation of ubiquitin pathways in dying flies. Activation of Wnt and glutathione may be mimicking the cardiac and neuronal abnormalities seen in both Drosophila and human situations [11,17,21,32,41].

Computational analysis of the upregulated genes in the 21-day group revealed a commonality in potential targeting by microRNA 315-5p that is evolutionarily conserved in *Drosophila* species. The genes involved are related to glutathione metabolism, sodium channel components, transcription factors, and genes with unknown function as of this writing [21,22,40]. Additionally, the down-regulated gene group at 21 days revealed multiple genes that could bind to miR-315-5p. These genes include GstE7, MtnD, CG14820, Lectin-galC1, kappaTry, among others. miR-315-5p has been frequently cited as playing a pivotal role in activating the Wg (wingless) pathway in *Drosophila*. Wg has an evolutionary relationship with Wnt in mammals [34]. Although the mechanism remains to be elucidated, the role of miR-315-5p is suspected of activating the Wg pathway by increasing the expression of Axin and Notum, both key proteins in controlling the canonical beta-catenin pathway [34]. Further, miR-315-5p and miR-8 play intertwining roles in Wg pathway activation [34,43]. Upon re-review of our gene expression database, miR-8 has come up less frequently to target genes in an evolutionarily conserved manner. There were no other evolutionarily conserved microRNAs in this study that came up as frequently. This is not to suggest that there are no microRNAs involved in sepsis and the response seen in *Drosophila*. Further in vitro studies would be needed to characterize these microRNAs binding to potential targets and assess for function in cell and animal models. Together, microRNAs are proposed to temper or amplify gene expression when needed rather than cause an absolute on or off expression pattern [1,13,41,43]. More importantly, the diverse clinical presentations of inflammation in sepsis and requirements of multiple factors suggest a role for closely tempering and amplifying gene products as needed; in this setting, microRNAs may play a key role.

The results of the study provide pilot data to support for Wg/Wnt involvement in post-sepsis homeostasis. Wg (Wingless) has been studied extensively in Drosophila and is arguably one of the most extensively studied intracellular signaling networks [19,36,43]. The function of Wg extends beyond body formation and involves controlling cytoplasmic levels of beta-catenin/Armadillo (Arm), differentiation of hematopoietic stem cells into B cells, and dendritic cell function [44,45]. Wnt signaling is an essential factor in microglia activation, rheumatoid arthritis, and the emergence of tumor-associated macrophages [46,47]. Finally, Wnt/beta-catenin signaling has been implicated in tissue remodeling, including in normal (wound healing) and pathological healing (lung fibrosis) [48,49]. 

A limitation of our study is the sole use of *E. coli* as the model of sepsis. Other models of sepsis might result in different findings as to the activation pattern change over time and are stimulus dependence [24,42,50]. To minimize environmental influence, we controlled the temperature and diet strictly, and samples were handled by very experienced staff. Furthermore, our study does not follow a typical clinical trajectory for humans where antibiotics would be employed early [7]. Our control group involved a sham surgery that provided confidence in the results as it removed the procedural element from the test group and variable. Finally, the gene expression was measured using RNA-seq. In vitro studies are needed to demonstrate the clinical importance of suggested miRNA induction of mRNA degradation as clinically relevant. Considering our report’s pilot and exploratory nature, we believe that future readers could browse the attached genomic database, identify, and verify targets of interest. Finally, one could question the clinical applicability of our study. The translational potential of each model of sepsis has to be questioned [6,17,25]. *Drosophila* may ideal to study mechanism of action in sepsis but not for immediate clinical bedside testing. The *Drosophila* model provides striking similarity at the level of metabolomic response, monocyte polarization activation of several innate immunity pathways [21,23,28,30,31,38,39,44,45]. Activation of these mechanisms translates into abnormalities in the cardiovascular and neuronal system similar to those seen in septic shock of mammalian models [2,10,17,22,30,32]. Drosophila models were instrumental in discovering several important immunological mechanisms and suggested future studies and treatments [1,2,17,22,25,28,31,37,40,44]. Most of the translation is indirect as the findings are fundamental and homologous in scientific and clinical nature [1,2,17,22,25,28,31,37,40,44]. Finally, the introduction of bacteria may mimic bacteremia but not sepsis [2,7,22,27]. The former is devoid of end-organ failure, but the immune system response is similar. Some of the prolonged immune abnormalities are triggered by the pathogen’s interaction with the host [25,26,27].

Our study demonstrates that changes in gene expression patterns extend long after the resolution of sepsis and are dynamic. This is an important fundamental finding that strengthens clinical observation of the prolonged effect of sepsis in survivors. Sepsis early in life triggered the expression of several transcriptional modules as the survivors age. Finally, our study suggests mRNA as one of the potential mechanisms affecting gene expression in sepsis survivors.

## 4. Materials and Methods

### 4.1. Fly Stock Maintenance and Experimental Procedure

Wild-type *Drosophila* stocks were maintained in standard corn meal–yeast agar medium vials at 25 °C. Seven to ten-day-old males were then inoculated in the thorax with concentrated *E. coli* pellets using a sterilized tungsten needle. The sham group was inoculated with a sterile phosphate buffer solution. Twenty flies were anesthetized with carbon dioxide and sacrificed at 7, 14, and 21 days following bacterial inoculation or sham surgery. 

### 4.2. RNA Extraction and Quantification

A total of 20 flies from each group (experimental and sham) were collected, weighed, and processed for RNA extraction at each time point. Five flies were pooled for each extraction, which was achieved through a spin column purification method (Zymo Research^®^, Orange, CA, USA). RNA purification was quantified using a spectrophotometer (Thermo Fisher Scientific, Waltham, MA, USA). Samples were defined as having acceptable purity with a 260/280 ratio greater than or equal to 2 using Nanodrop.

### 4.3. RNA Sequencing and Analysis

Before the library building, the samples underwent the quality check utilizing agarose electrophoresis and RNA integrity checks on a Agilent 2100 platform. mRNA was enriched using oligo(dT) beads, followed by random fragmentation and cDNA synthesis using random hexamers and reverse transcriptase. After first-strand synthesis, a custom second-strand synthesis buffer was added with dNTP, RNAase H, and *E. coli* polymerase I to generate the second strand by nick translation. The final cDNA library was ready after additional purification, terminal repair, A-tailing, ligation of sequencing adapter, size selection, and PCR enrichment. 

Library concentration was quantified using Qubit 2.0 fluorometer (Life Technologies, Carlsbad, CA, USA) and adjusted to 1 ng/uL before checking insert size on the Agilent 2100 platform and quantitative PCR with the goal of library activity >2 nM. RNA sequencing was performed using an Illumina next-generation sequencing platform. 

The original raw data were transformed to sequenced reads by base calling. Raw data were recorded in the FASTQ file. The error rate for each base was calculated as a Phred score. The quality of data was analyzed by A/T/G/C content distribution. Finally, the data were filtered to discard reads with adaptor contamination, uncertain nucleotides more than 10%, and low-quality nucleotides, defined as base quality less than 20 constituting more than 50% of reads. 

Bowtie2 was chosen for mapping the reference genome. The mismatch parameter was set to two, with other parameters as default. Only filtered reads were used to analyze the mapping of RNA-seq to the reference genome. Total Mapped Reads of Fragments was larger than 70%, and Multiple Mapped Reads or Fragments was below 10%. Mapped regions were classified as exons, introns, or intergenic regions. 

After mapping to the reference genome, gene expression levels were quantified by measuring transcript abundance. HTSeq software was used in union mode. Differential gene expression was done after read-count normalization, model-dependent *p*-value estimation, and FDR value estimation using multiple hypothesis testing; padj of less than 0.05 was utilized as we had replicates. GO ontology was conducted using GOseq to analyze differentially expressed genes;these genes were utilized to create KEGG pathways. 

### 4.4. 3′ Untranslated Region and microRNA Analysis

The gene lists generated from HTSeq software were analyzed using ENSEMBL gene browser for 3′ untranslated region. The list of genes were analyzed individually and in batches using TargetScanFly (TargetScanFly 6.2). Potential targets were analyzed using the database software for candidate microRNAs. Additional information about candidate microRNAs was evaluated using RNA22 (RNA22|MicroRNA Target Predictions|Computational Medicine Center at Jefferson University). A further 3′ untranslated region analysis was also done using miRFocus (microRNA 2007—Predict your UTR (weizmann.ac.il)).

### 4.5. Statistical Analysis

The Shapiro–Wilk W test and distribution plots were used to test the normality of distribution variables. Parametric variables will be expressed as mean ± SD and compared using the *t*-Student test. Statistical analyses was performed with the Statistica 11.0 (StatSoft Inc., Tulsa, OK, USA).

## 5. Conclusions

The temporal changes in gene expression in the *Drosophila* survivors of sepsis had distinct patterns in the long term after sepsis resolution. 

## Data Availability

The RNA-seq data will be deposited in FlyBase.

## Supplementary Materials

The following are available online at https://www.mdpi.com/article/10.3390/ijms22115422/s1, Figure S1: Activation pathways in 14 days sepsis survivors, Table S1: RNA-seq workflow.

## References

[1] Vachharajani V., McCall C.E. (2019). Epigenetic and metabolic programming of innate immunity in sepsis. Innate Immun..

[2] Nowill A.E., Fornazin M.C., Spago M.C., Neto V.D., Pinheiro V.R.P., Alexandre S.S.S., Moraes E.O., Souza G.H.M.F., Eberlin M.N., Marques L.A. (2019). Immune Response Resetting in Ongoing Sepsis. J. Immunol..

[3] Mankowski R.T., Yende S., Angus D.C. (2019). Long-term impact of sepsis on cardiovascular health. Intensiv. Care Med..

[4] Abu-Kaf H., Mizrakli Y., Novack V., Dreiher J. (2018). Long-Term Survival of Young Patients Surviving ICU Admission with Severe Sepsis. Crit. Care Med..

[5] Shen H.-N., Lu C.-L., Yang H.-H. (2016). Risk of Recurrence after Surviving Severe Sepsis. Crit. Care Med..

[6] Coopersmith C.M., De Backer D., Deutschman C.S., Ferrer R., Lat I., Machado F.R., Martin G.S., Martin-Loeches I., Nunnally M.E., Antonelli M. (2018). Surviving sepsis campaign: Research priorities for sepsis and septic shock. Intensiv. Care Med..

[7] Weiss S.L., Peters M.J., Alhazzani W., Agus M.S.D., Flori H.R., Inwald D.P., Nadel S., Schlapbach L.J., Tasker R.C., Argent A.C. (2020). Surviving sepsis campaign international guidelines for the management of septic shock and sepsis-associated organ dysfunction in children. Intensiv. Care Med..

[8] Nascimento D.C., Melo P.H., Piñeros A.R., Ferreira R.G., Colón D.F., Donate P.B., Castanheira F.V., Gozzi A., Czaikoski P.G., Niedbala W. (2017). IL-33 contributes to sepsis-induced long-term immunosuppression by expanding the regulatory T cell population. Nat. Commun..

[9] Lapko N., Zawadka M., Polosak J., Worthen G.S., Danet-Desnoyers G., Puzianowska-Kuźnicka M., Laudanski K. (2017). Long-term Monocyte Dysfunction after Sepsis in Humanized Mice Is Related to Persisted Activation of Macrophage-Colony Stimulation Factor (M-CSF) and Demethylation of PU.1, and It Can Be Reversed by Blocking M-CSF In Vitro or by Transplanting Naïve Autologous Stem Cells In Vivo. Front. Immunol..

[10] Kaynar A.M., Bakalov V., LaVerde S.M., Cambriel A.I.F., Lee B.-H., Towheed A., Gregory A.D., Webb S.A.R., Palladino M.J., Bozza F.A. (2016). Cost of surviving sepsis: A novel model of recovery from sepsis in Drosophila melanogaster. Intensiv. Care Med. Exp..

[11] Brakenridge S.C., Efron P.A., Cox M.C., Stortz J.A., Hawkins R.B., Ghita G., Gardner A., Mohr A.M., Anton S.D., Moldawer L.L. (2019). Current Epidemiology of Surgical Sepsis. Ann. Surg..

[12] Bomans K., Schenz J., Sztwiertnia I., Schaack D., Weigand M.A., Uhle F. (2018). Sepsis Induces a Long-Lasting State of Trained Immunity in Bone Marrow Monocytes. Front. Immunol..

[13] Fouda E., Midan D.A.E., Ellaban R., El-Kousy S., Arafat E. (2021). The diagnostic and prognostic role of MiRNA 15b and MiRNA 378a in neonatal sepsis. Biochem. Biophys. Rep..

[14] Tang Y., Yang X., Shu H., Yu Y., Pan S., Xu J., Shang Y. (2021). Bioinformatic analysis identifies potential biomarkers and therapeutic targets of septic-shock-associated acute kidney injury. Hereditas.

[15] Haas L.E.M., Boumendil A., Flaatten H., Guidet B., Ibarz M., Jung C., Moreno R., Morandi A., Andersen F.H., Zafeiridis T. (2021). Frailty is associated with long-term outcome in patients with sepsis who are over 80 years old: Results from an observational study in 241 European ICUs. Age Ageing.

[16] Jeansonne D., Jeyaseelan S. (2021). Role of an Anti-Aging Molecule in a Toxic Lifestyle: Relevance for Alcohol Effects on Sepsis. Alcohol. Clin. Exp. Res..

[17] Popov D., Pavlov G. (2013). Sepsis models in exprimental animals. Trakia J. Sci..

[18] Cho S.-Y., Kwon Y.-K., Nam M., Vaidya B., Kim S.R., Lee S., Kwon J., Kim D., Hwang G.-S. (2017). Integrated profiling of global metabolomic and transcriptomic responses to viral hemorrhagic septicemia virus infection in olive flounder. Fish Shellfish Immunol..

[19] Douglas A.E. (2018). The Drosophila model for microbiome research. Lab Anim..

[20] Medzhitov R., Preston-Hurlburt P., Janewayr C.A. (1997). A human homologue of the Drosophila Toll protein signals activation of adaptive immunity. Nat. Cell Biol..

[21] Medvedev A.E., Murphy M.P., Zhou H., Li X. (2015). E3 ubiquitin ligases Pellinos as regulators of pattern recognition receptor signaling and immune responses. Immunol. Rev..

[22] Istas O., Greenhalgh A., Cooper R. (2019). The Effects of a Bacterial Endotoxin on Behavior and Sensory-CNS-Motor Circuits in Drosophila melanogaster. Insects.

[23] Limmer S., Haller S., Drenkard E., Lee J., Yu S., Kocks C., Ausubel F.M., Ferrandon D. (2011). Pseudomonas aeruginosa RhlR is required to neutralize the cellular immune response in a Drosophila melanogaster oral infection model. Proc. Natl. Acad. Sci. USA.

[24] Ramond E., Dudzic J.P., Lemaitre B. (2020). Comparative RNA-Seq analyses of Drosophila plasmatocytes reveal gene specific signatures in response to clean injury and septic injury. PLoS ONE.

[25] Heo Y.-J., Lee Y.-R., Jung H.-H., Lee J., Ko G., Cho Y.-H. (2009). Antibacterial Efficacy of Phages against Pseudomonas aeruginosa Infections in Mice and Drosophila melanogaster. Antimicrob. Agents Chemother..

[26] Gotland N., Uhre M., Mejer N., Skov R., Petersen A., Larsen A., Benfield T. (2016). Long-term mortality and causes of death associated with Staphylococcus aureus bacteremia. A matched cohort study. J. Infect..

[27] Artero A., Inglada L., Gómez-Belda A., Capdevila J.A., Diez L.F., Arca A., Romero J.M., Domínguez-Gil M., Serra-Centelles C., De La Fuente J. (2018). The clinical impact of bacteremia on outcomes in elderly patients with pyelonephritis or urinary sepsis: A prospective multicenter study. PLoS ONE.

[28] Matova N., Anderson K.V. (2006). Rel/NF- B double mutants reveal that cellular immunity is central to Drosophila host defense. Proc. Natl. Acad. Sci. USA.

[29] Chen J., Xie C., Tian L., Hong L., Wu X., Han J. (2010). Participation of the p38 pathway in Drosophila host defense against pathogenic bacteria and fungi. Proc. Natl. Acad. Sci. USA.

[30] Avet-Rochex A., Perrin J., Bergeret E., Fauvarque M.-O. (2007). Rac2 is a major actor of Drosophila resistance to Pseudomonas aeruginosa acting in phagocytic cells. Genes Cells.

[31] Bajgar A., Krejčová G., Doležal T. (2021). Polarization of Macrophages in Insects: Opening Gates for Immuno-Metabolic Research. Front. Cell Dev. Biol..

[32] Bakalov V., Amathieu R., Triba M.N., Clément M.-J., Uribe L.R., Le Moyec L., Kaynar A.M. (2016). Metabolomics with Nuclear Magnetic Resonance Spectroscopy in a Drosophila melanogaster Model of Surviving Sepsis. Metabolites.

[33] Irving P., Ubeda J.-M., Doucet D., Troxler L., Lagueux M., Zachary D., Hoffmann J.A., Hetru C., Meister M. (2005). New insights into Drosophila larval haemocyte functions through genome-wide analysis. Cell. Microbiol..

[34] Silver S.J., Hagen J.W., Okamura K., Perrimon N., Lai E.C. (2007). Functional screening identifies miR-315 as a potent activator of Wingless signaling. Proc. Natl. Acad. Sci. USA.

[35] Singh S.P., Coronella J.A., Beneš H., Cochrane B.J., Zimniak P. (2001). Catalytic function ofDrosophila melanogasterglutathioneS-transferase DmGSTS1-1 (GST-2) in conjugation of lipid peroxidation end products. JBIC J. Biol. Inorg. Chem..

[36] Yan J., Ralston M.M., Meng X., Bongiovanni K.D., Jones A.L., Benndorf R., Nelin L.D., Frazier W.J., Rogers L.K., Smith C.V. (2013). Glutathione reductase is essential for host defense against bacterial infection. Free Radic. Biol. Med..

[37] Udomsinprasert R., Bogoyevitch M.A., Ketterman A.J. (2004). Reciprocal regulation of glutathione S-transferase spliceforms and the Drosophila c-Jun N-terminal kinase pathway components. Biochem. J..

[38] Wongtrakul J., Sukittikul S., Saisawang C., Ketterman A.J. (2012). Mitogen-Activated Protein Kinase p38b Interaction with Delta Class Glutathione Transferases from the Fruit Fly, Drosophila melanogaster. J. Insect Sci..

[39] Ekas L.A., Baeg G.-H., Flaherty M.S., Ayala-Camargo A., Bach E.A. (2006). JAK/STAT signaling promotes regional specification by negatively regulating wingless expression in Drosophila. Development.

[40] Mockett R.J., Sohal R.S., Orr W.C. (1999). Overexpression of glutathione reductase extends survival in transgenic Drosophila melanogaster under hyperoxia but not normoxia. FASEB J..

[41] Missirlis F., Rahlfs S., Dimopoulos N., Bauer H., Becker K., Hilliker A., Phillips J.P., Jäckle H. (2003). A Putative Glutathione Peroxidase of Drosophila Encodes a Thioredoxin Peroxidase That Provides Resistance against Oxidative Stress But Fails to Complement a Lack of Catalase Activity. Biol. Chem..

[42] Silveira-Lessa A.L., Quinello C., Lima L., Redondo A.C.C., Ceccon M.E.J.R., Carneiro-Sampaio M., Palmeira P. (2016). TLR expression, phagocytosis and oxidative burst in healthy and septic newborns in response to Gram-negative and Gram-positive rods. Hum. Immunol..

[43] Kennell J.A., Gerin I., MacDougald O.A., Cadigan K.M. (2008). The microRNA miR-8 is a conserved negative regulator of Wnt signaling. Proc. Natl. Acad. Sci. USA.

[44] Jridi I., Canté-Barrett K., Pike-Overzet K., Staal F.J.T. (2021). Inflammation and Wnt Signaling: Target for Immunomodulatory Therapy?. Front. Cell Dev. Biol..

[45] Kwee S.A., Tiirikainen M. (2021). Beta-catenin activation and immunotherapy resistance in hepatocellular carcinoma: Mechanisms and biomarkers. Hepatoma Res..

[46] Claudel M., Jouzeau J., Cailotto F. (2019). Secreted Frizzled-related proteins (sFRPs) in osteo-articular diseases: Much more than simple antagonists of Wnt signaling?. FEBS J..

[47] Rastogi M., Srivastava N., Singh S.K. (2018). Exploitation of microRNAs by Japanese Encephalitis virus in human microglial cells. J. Med. Virol..

[48] Wang Y., Dong X., Zhao N., Su X., Wang Y., Li Y., Wen M., Li Z., Wang C., Chen J. (2020). Schisandrin B attenuates bleomycin-induced pulmonary fibrosis in mice through the wingless/integrase-1 signaling pathway. Exp. Lung Res..

[49] Blankesteijn W.M. (2019). Interventions in WNT Signaling to Induce Cardiomyocyte Proliferation: Crosstalk with Other Pathways. Mol. Pharmacol..

[50] Grealy R., White M., Stordeur P., Kelleher D., Doherty D.G., McManus R., Ryan T. (2013). Characterising Cytokine Gene Expression Signatures in Patients with Severe Sepsis. Mediat. Inflamm..

