# Supplementary material for: Prolonged Transcriptional Consequences in Survivors of Sepsis
Source: Int J Mol Sci. 2021 May 21;22(11):5422. doi: 10.3390/ijms22115422 (PMC8196560; doi:10.3390/ijms22115422)

Supplementary Materials:

Figure S1: Activation pathways in 14 days sepsis survivors

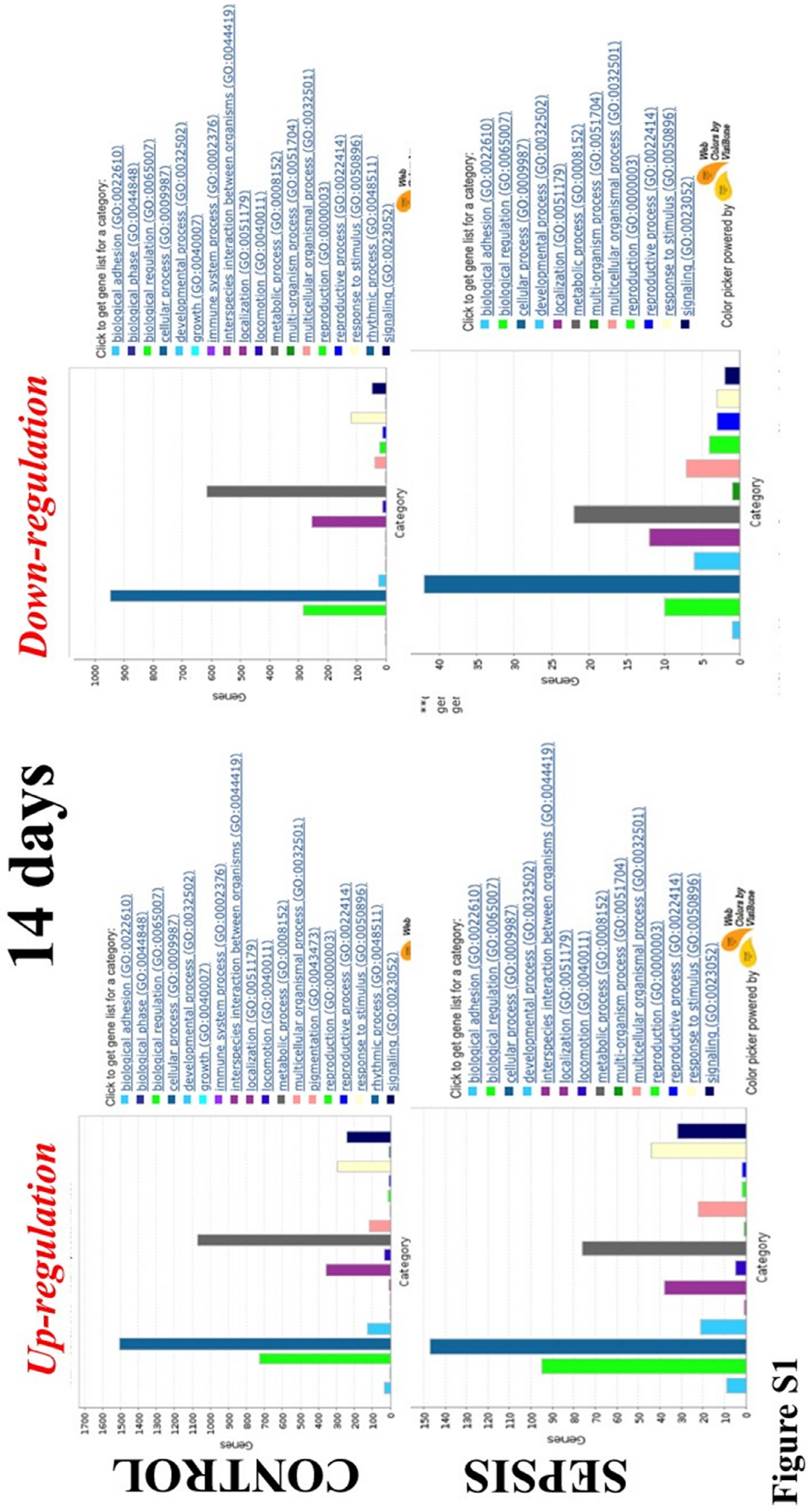

Figure S2: Activation pathways in 21 days sepsis survivors

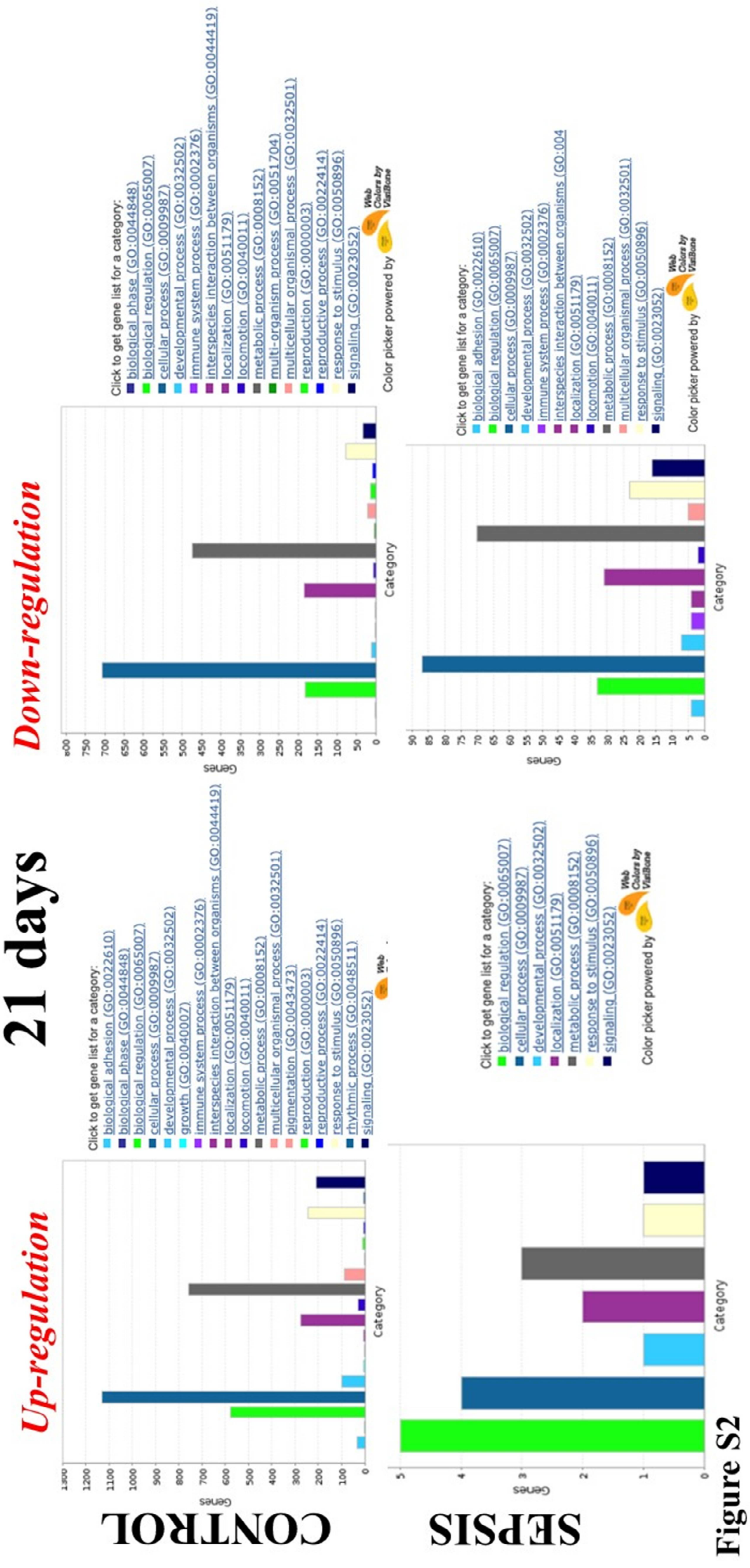

Figure S3: RNA-seq workflow.

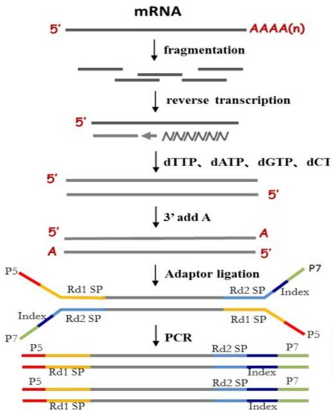

Figure S1

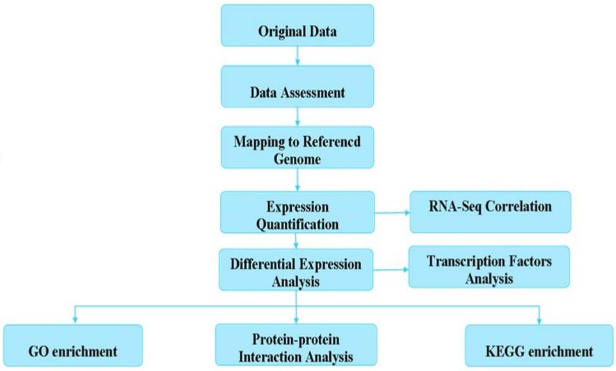

Supplement: Supplementary file 1 [file ijms-22-05422-s001.zip › ijms-1214102-supplementary.pdf]
